# Association Between Nutritional Status and Activities of Daily Living in Super-elderly Patients With Proximal Femoral Fractures After Surgery in a Rehabilitation Ward During the Convalescent Phase

**DOI:** 10.7759/cureus.95727

**Published:** 2025-10-30

**Authors:** Tomoko Ota, Yaeko Oikawa, Kazuhiro Toyama, Shuji Matsumoto

**Affiliations:** 1 Department of Rehabilitation Medicine, Sakura-Koseien Hospital, Sakura, JPN; 2 Department of Medical Technology, Sakura-Koseien Hospital, Sakura, JPN; 3 Department of Internal Medicine, Sakura-Koseien Hospital, Sakura, JPN; 4 Center for Medical Sciences, Ibaraki Prefectural University of Health Sciences, Ami-cho, JPN

**Keywords:** activities of daily living (adls), convalescent rehabilitation, nutritional management, proximal femur fracture, super-elderly

## Abstract

Background

This study aimed to investigate the relationship between nutritional status and improvement in activities of daily living (ADLs) in super-elderly patients with proximal femoral fractures who underwent surgery in a rehabilitation ward during the convalescent phase.

Methodology

A retrospective study was conducted among patients with proximal femoral fractures who were discharged from the convalescent rehabilitation ward of Sakura-Koseien Hospital. This study included 21 patients aged 90 years or older and 43 patients aged 75-89 years. Nutritional assessment items included the Mini Nutritional Assessment-Short Form (MNA-SF) at admission and calf circumference. Body mass index (BMI), serum albumin (Alb), Controlling Nutritional Status (CONUT), and Geriatric Nutritional Risk Index (GNRI) were extracted from the medical records at admission, one month after admission, and at the final evaluation. Furthermore, the correlation between Functional Independence Measure (FIM) gain (difference in the total score of the FIM motor items between admission and discharge) and nutritional assessment items was investigated.

Results

In the super-elderly group, the correlation between each nutritional assessment item at admission and FIM gain was as follows: MNA-SF (p = 0.533), lower leg circumference (p = 0.300), BMI (p = 0.440), Alb (p = 0.050), CONUT (p = 0.129), and GNRI (p = 0.192). No significant correlations were found (p < 0.05). One month after admission, CONUT (correlation coefficient (r) = -0.587, confidence interval (CI) = -0.813 to -0.209) and Alb (r = 0.505, CI = 0.093 to 0.769) showed correlations with FIM improvement. At the final evaluation, CONUT (r = -0.525, CI = -0.780 to -0.121) and Alb (r = 0.466, CI = 0.043 to 0.747) showed a significant correlation with FIM improvement (all p < 0.05).

Conclusions

In super-elderly patients with proximal femoral fractures, the CONUT score and Alb levels one month after hospitalization were suggested to be associated with improvement in ADLs.

## Introduction

Because of the super-aged society, it is estimated that by 2040, the population aged 65 years and over in Japan will account for approximately 35% of the total population of 112.84 million [1]. According to the 2021 Japanese Guidelines for the Management of Femoral Neck/Trochanteric Fractures (revised third edition), the annual number of new patients with femoral neck/trochanteric fractures is estimated to reach 320,000 by 2040 [2]. Even among patients aged ≥90 years, who are generally considered to be super-elderly, the number of hip fractures is projected to increase to 146,000 by 2040 [3].

According to the results of the National Health and Nutrition Survey conducted by the Ministry of Health, Labour and Welfare, the proportion of elderly individuals aged ≥65 years with malnutrition (body mass index (BMI) ≤20 kg/m²), if categorized by age group, is the highest among those aged ≥85 years, with a high proportion of individuals exhibiting malnutrition [4]. Conversely, the effectiveness of rehabilitation for patients who have undergone surgery for proximal femoral fractures is influenced by factors such as the patient’s age and the presence of nutritional deficiencies; nutritional status is associated with improvements in activities of daily living (ADLs) [3,5,6].

Previous studies have reported that nutritional status and ADLs are associated with proximal femoral fractures postoperatively [7-9]. There are reports that nutritional status at admission to a rehabilitation ward during the convalescent phase is associated with ADLs at admission and discharge [10-12]. Furthermore, although it is recognized that malnutrition can significantly impact ADL outcomes [5], there are few reports focusing on the nutritional status and ADLs during the convalescent phase of patients with proximal femur fractures, whose numbers are expected to increase eventually, particularly in the super-elderly population.

Therefore, this study aimed to investigate the association between nutritional status and changes in ADLs following rehabilitation treatment in super-elderly patients with proximal femur fractures admitted to a convalescent rehabilitation ward. We aimed to determine which time-point of nutritional assessment, at admission, one month, or final evaluation, best correlates with ADL gains in super-elderly patients (≥90 years) undergoing convalescent rehabilitation after proximal femoral fracture surgery. We hypothesized that nutritional indicators after hospitalization would show a stronger correlation than those at admission.

## Materials and methods

Participants

This study consecutively enrolled patients aged ≥75 years who underwent surgery for proximal femoral fractures and were discharged from the rehabilitation ward of our hospital between April 2020 and March 2023. Patients with multiple fractures, those who were transferred to another hospital because of reoperation or treatment for other diseases, those who were readmitted to our hospital during the study period because of refracture, and those with missing data were excluded. Among the participants, patients aged ≥90 years were classified into the super-elderly group, and those aged 75-89 years were classified into the control group. Participant selection was based on the medical records. This study was conducted in accordance with the Declaration of Helsinki and approved by the Ethics Review Committee of our institution (approval number: 23-16; date of approval: April 21, 2023).

Research procedure and outcome measurement

This retrospective study examined the correlation between nutritional assessment items and ADL indicators in the super-elderly and control groups. Nutritional assessment items were selected based on those regularly evaluated at our institution, and included the Mini Nutritional Assessment-Short Form (MNA-SF) [13], calf circumference, BMI, serum albumin level (Alb), Controlling Nutritional Status (CONUT) [14], and Geriatric Nutritional Risk Index (GNRI) [15]. The MNA-SF and calf circumference were assessed upon admission. For items assessed monthly (BMI, Alb, CONUT, and GNRI), the results from the time of admission, one month after admission, and at the final assessment were used. The final evaluation was defined as the most recent regular assessment closest to the date of discharge from our hospital. The primary outcome was improvement in the Functional Independence Measure (FIM) motor gain from admission to discharge, and the main explanatory variables were the nutritional indices at admission, at one month, and at final evaluation. The ADL index was calculated as the FIM gain (discharge score minus admission score) using the total FIM motor item scores at admission and discharge from the hospital’s convalescent rehabilitation ward. The values for each evaluation item were extracted from the medical records and calculated. Blood biochemical data were obtained upon admission and during routine monthly examinations. The GNRI calculation formula is GNRI = {14.89 × Alb value (g/dL)} + {41.7 × actual body weight (kg)/ideal body weight (kg)}, in which ideal body weight is calculated using Lorenz’s formula; for men, height (cm) - 100 - {(height (cm) - 150)/4}, and for women, height (cm) - 100 - {(height (cm) - 150)/2.5} [16]. We have confirmed that the measurement instruments, scores, and indicators used in this study are freely available for use and do not require any license or permission.

Statistical analysis

As background information of the participants, we compared the following variables between the super-elderly and control groups using the Mann-Whitney U test: number of days from surgery to admission to our hospital, length of hospital stay, number of patients discharged to their homes [17], nutritional assessment items upon admission, total FIM motor item scores upon admission, total FIM cognitive item scores upon admission, and FIM gain. Chi-square tests were used for statistical analysis of sex and fracture type [18]. Additionally, within each group (ultra-elderly and control), we analyzed the correlation between each nutritional assessment item and FIM gain using Spearman’s rank correlation coefficient. As some variables were non-normally distributed, we standardized the statistical analysis using nonparametric tests. In all statistical analyses, the significance level was set at 5% and the analysis software used was modified R Commander 4.3.1.

## Results

**Figure 1:**
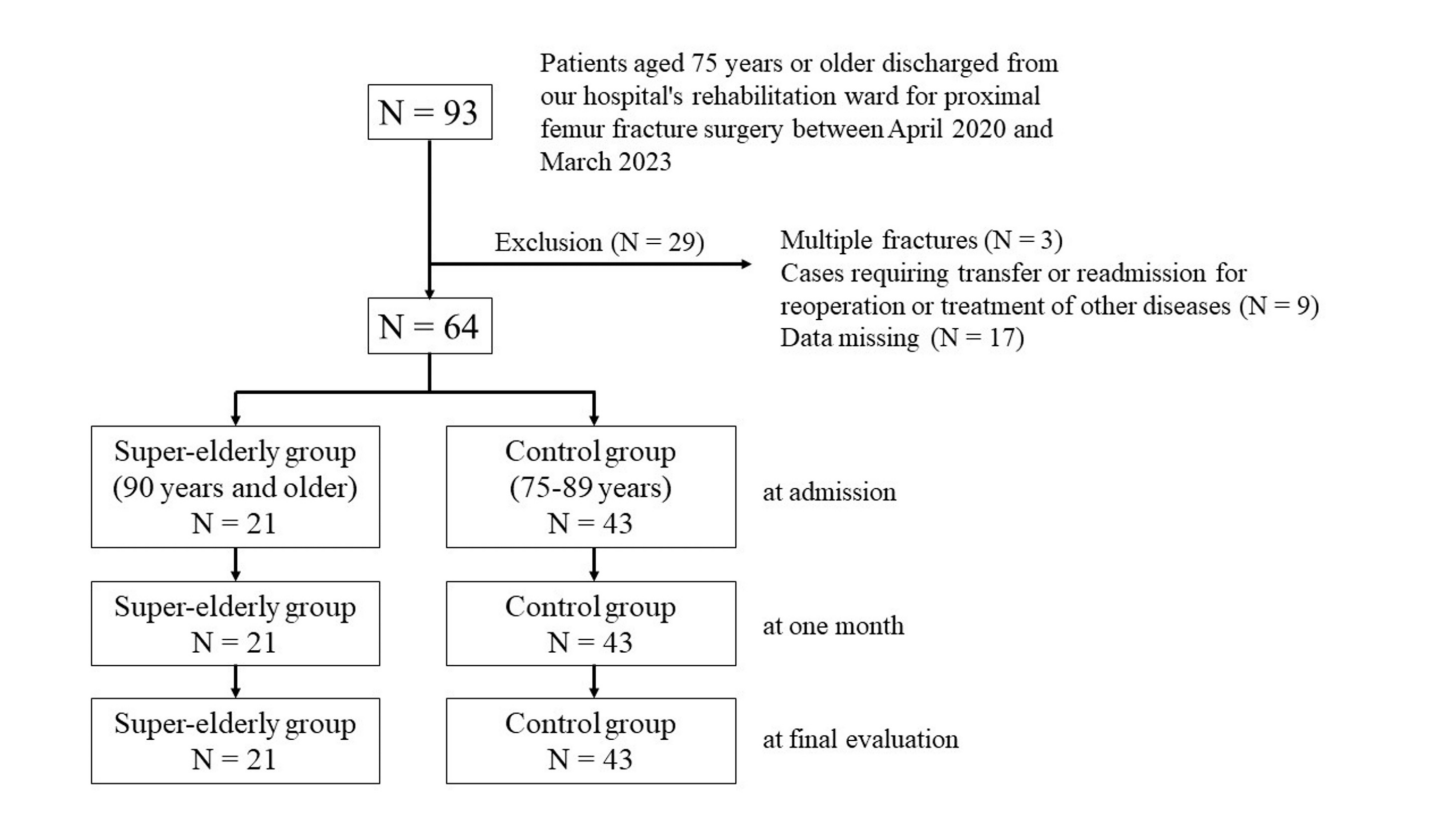
Flowchart of the clinical trial.

Of the 82 patients aged ≥75 years who were discharged from our hospital’s rehabilitation ward for the convalescent phase between April 2020 and March 2023 following surgery for proximal femur fractures, 64 met the selection criteria. Among these, 21 patients (three men and 18 women) were in the super-elderly group aged ≥90 years, and the control group aged 75-89 years included 43 patients (16 men and 27 women) (Figure 1). The median age was 92 (interquartile range (IQR) = 91-94) years in the super-elderly group and 84 (IQR = 80-86.5) years in the control group, respectively. The fracture types were as follows: in the super-elderly group, nine patients had femoral neck fractures and 12 patients had trochanteric fractures; in the control group, 22 patients had femoral neck fractures and 21 patients had trochanteric fractures. The median number of days from surgery to hospitalization was 21 (IQR = 17-34) days in the super-elderly group and 23 (IQR = 19-32.5) days in the control group. The median length of hospital stay was 66 (IQR = 53-80) days in the super-elderly group and 74 (IQR = 59.5-81.5) days in the control group. The average number of days from surgery to admission was 24.9 ± 14.2 and 26.8 ± 11.1 days in the super-elderly and control groups, respectively. The average length of hospital stay was 66.3 ± 15.8 and 70.0 ± 15.0 days in the super-elderly and control groups, respectively. The number of patients discharged to home was 11 and 34 in the super-elderly and control groups, respectively (Table 1). All patients in both groups were able to eat orally. Additionally, the results of the Mann-Whitney U test, median values of the FIM gain, total scores of the FIM motor items upon admission, total scores of the FIM cognitive items upon admission, and nutritional assessment items upon admission are presented in Table 1. Significant differences were observed between the two groups regarding the number of patients discharged to their homes, calf circumference, GNRI score, total FIM motor item scores, and total FIM cognitive item scores at admission.

**Table 1:** Participant characteristics. *: Values are expressed as median (interquartile range, IQR). MNA-SF = Mini Nutritional Assessment-Short Form; BMI = body mass index; Alb = serum albumin; CONUT = Controlling Nutritional Status; GNRI = Geriatric Nutritional Risk Index; FIM = Functional Independence Measure

	Super-elderly group (n = 21)	Control group (n = 43)	P-value
Age (years)*	92 (91–94)	84 (80–86.5)	<0.001
Gender (n)			0.059
Male	3	16	
Female	18	27	
Types of fractures			0.533
Postoperative femoral neck fracture (n)	9	22	
Right	4	8	
Left	5	14	
Postoperative femoral trochanteric fracture (n)	12	21	-
Right	7	10	
Left	5	11	
Days from surgery to admission at our hospital (days)*	21 (17–34)	23 (19–32.5)	0.386
Length of hospital stay (days)*	66 (53–80)	74 (59.5–81.5)	0.44
Number of discharges to home (n)	11	34	0.029
MNA-SF at admission (points)*	6.0 (5.0–7.0)	6.0 (5.5–7.5)	0.207
Calf circumference at admission (cm)*	27.0 (25.0–29.0)	29.8 (27.9–32.5)	0.003
BMI at admission (kg/m^2^)*	19.4 (17.9–21.0)	21.1 (18.7–22.7)	0.133
Alb at admission (g/dL)*	3.3 (3.0–3.8)	3.6 (3.3–3.8)	0.078
CONUT at admission (score)*	3.0 (2.0–5.0)	3.0 (2.0–4.0)	0.228
GNRI at admission*	86.6 (82.2–91.9)	92.2 (85.4–97.0)	0.027
FIM movement item total at admission (points)*	36.0 (26.0–40.0)	45.0 (33.0–51.0)	0.001
FIM cognitive item total at admission (points)*	18.0 (16.0 ± 21.0)	22.0 (16.5 ± 29.0)	0.015
FIM gains (points)*	19.0 (10.0–27.0)	25.0 (16.0–29.5)	0.195

The median values of the evaluation items for each group are presented in Table 2. The median MNA-SF score at admission was 6.0 (5.0-7.0) and 6.0 (5.5-7.5) points in the super-elderly and control groups, respectively. The median calf circumference upon admission was 27.0 (25.0-29.0) and 29.8 (27.9-32.5) cm in the super-elderly and control group, respectively. BMI upon admission was 19.4 (17.9-21.0) and 21.1 (18.7-22.7) kg/m² in the super-elderly and control groups, respectively. Albumin levels were 3.3 (3.0-3.8) and 3.6 (3.3-3.8) g/dL in the super-elderly and control groups, respectively. CONUT scores were 3.0 (2.0-5.0) and 3.0 (2.0-4.0) points in the super-elderly and control groups, respectively. GNRI was 86.6 (82.2-91.9) and 92.2 (85.4-97.0) in the super-elderly and control groups, respectively. The total score for the FIM motor items was 36.0 (26.0-40.0) and 45 (33.0-51.0) points in the super-elderly and control groups, respectively. One month after hospitalization, BMI was 19.1 (17.9-20.5) and 20.2 (18.5-22.1) kg/m² in the super-elderly and control groups, respectively. The albumin level was 3.3 (3.0-3.5) and 3.7 (3.5-4.0) g/dL in the super-elderly and control group, respectively. CONUT was 3.0 (2.0-5.0) and 2.0 (1.0-3.0) points, and GNRI was 84.2 (79.8-90.7) and 93.2 (86.0-99.0) in the super-elderly and control groups, respectively. At the final evaluation, BMI was 19.1 (17.8-21.5) and 20.3 (18.7-22.0) kg/m² in the super-elderly and control groups, respectively. Alb levels were 3.3 (3.1-3.8) and 3.7 (3.5-4.0) g/dL in the super-elderly and control groups, respectively. CONUT was 3.0 (2.0-4.0) and 2.0 (1.0-3.0), and GNRI was 86.1 (80.3-90.9) and 94.2 (86.7-98.9) in the super-elderly and control groups, respectively. The FIM motor score was 55.0 (42.0-64.0) and 73 (54.0-79.0) points in the super-elderly and control groups, respectively. The FIM gain was 19.0 (10.0-27.0) and 25.0 (16.0-29.5) points in the super-elderly and control groups, respectively.

**Table 2:** Values of evaluation items at admission, one month after admission, and at the final evaluation. All values are expressed as median (interquartile range, IQR). MNA-SF = Mini Nutritional Assessment-Short Form; BMI = body mass index; Alb = serum albumin; CONUT = Controlling Nutritional Status; GNRI = Geriatric Nutritional Risk Index; FIM = Functional Independence Measure

	Evaluation items	Super-elderly group (n = 21)	Control group (n = 43)
At admission	MNA-SF (points)	6.0 (5.0–7.0)	6.0 (5.5–7.5)
	Calf circumference (cm)	27.0 (25.0–29.0)	29.8 (27.9–32.5)
	BMI (kg/m^2^)	19.4 (17.9–21.0)	21.1 (18.7–22.7)
	Alb (g/dL)	3.3 (3.0–3.8)	3.6 (3.3–3.8)
	CONUT (score)	3.0 (2.0–5.0)	3.0 (2.0–4.0)
	GNRI	86.6 (82.2–91.9)	92.2 (85.4–97.0)
	FIM movement item total at admission (points)	36.0 (26.0–40.0)	45.0 (33.0–51.0)
At one month	BMI (kg/m^2^)	19.1 (17.9–20.5)	20.2 (18.5–22.1)
	Alb (g/dL)	3.3 (3.0–3.5)	3.7 (3.5–4.0)
	CONUT (score)	3.0 (2.0–5.0)	2.0 (1.0–3.0)
	GNRI	84.2 (79.8–90.7)	93.2 (86.0–99.0)
At the final evaluation	BMI (kg/m^2^)	19.1 (17.8–21.5)	20.3 (18.7–22.0)
	Alb (g/dL)	3.3 (3.1–3.8)	3.7 (3.5-4.0)
	CONUT (score)	3.0 (2.0–4.0)	2.0 (1.0–3.0)
	GNRI	86.1 (80.3–90.9)	94.2 (86.7–98.9)
	FIM movement item total at admission (points)	55.0 (42.0–64.0)	73.0 (54.0–79.0)
	FIM gains (points)	19.0 (10.0–27.0)	25.0 (16.0–29.5)

**Figure 2:**
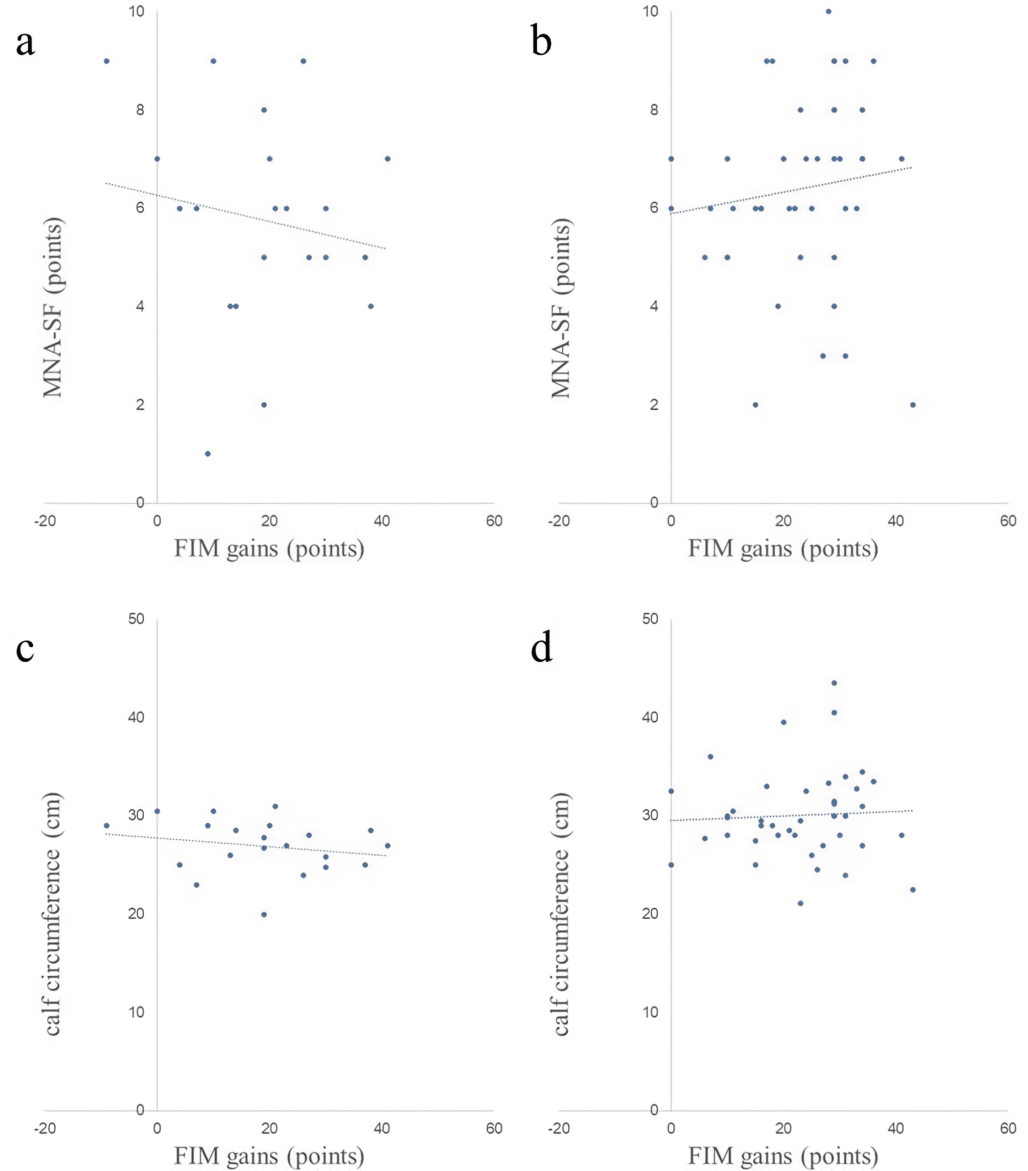
Scatter plot of nutritional evaluation items (MNA-SF and calf circumference) at admission and FIM gains. (a) MNA-SF at admission and FIM gains in the super-elderly group. (b) MNA-SF at admission and FIM gains in the control group. (c) calf circumference at admission and FIM gains in the super-elderly group. (d) calf circumference at admission and FIM gains in the control group. MNA-SF = Mini Nutritional Assessment-Short Form; FIM = Functional Independence Measure

**Figure 3:**
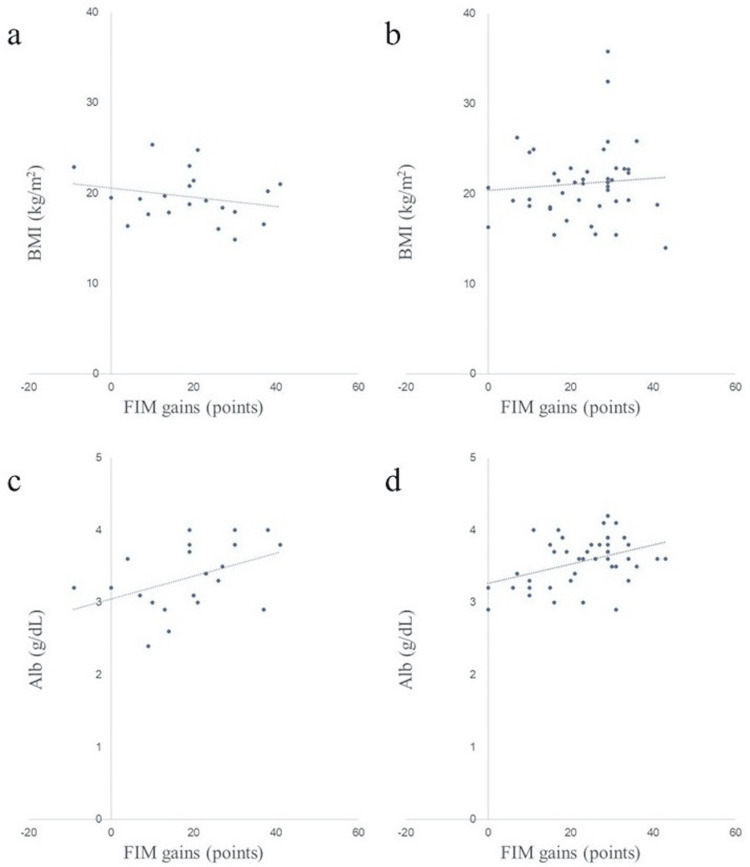
Scatter plot of nutritional evaluation items (BMI and Alb) at admission and FIM gains. (a) BMI at admission and FIM gains in the super-elderly group. (b) BMI at admission and FIM gains in the control group. (c) Alb at admission and FIM gains in the super-elderly group. (d) Alb at admission and FIM gains in the control group. BMI = body mass index; Alb = serum albumin; FIM = Functional Independence Measure

**Figure 4:**
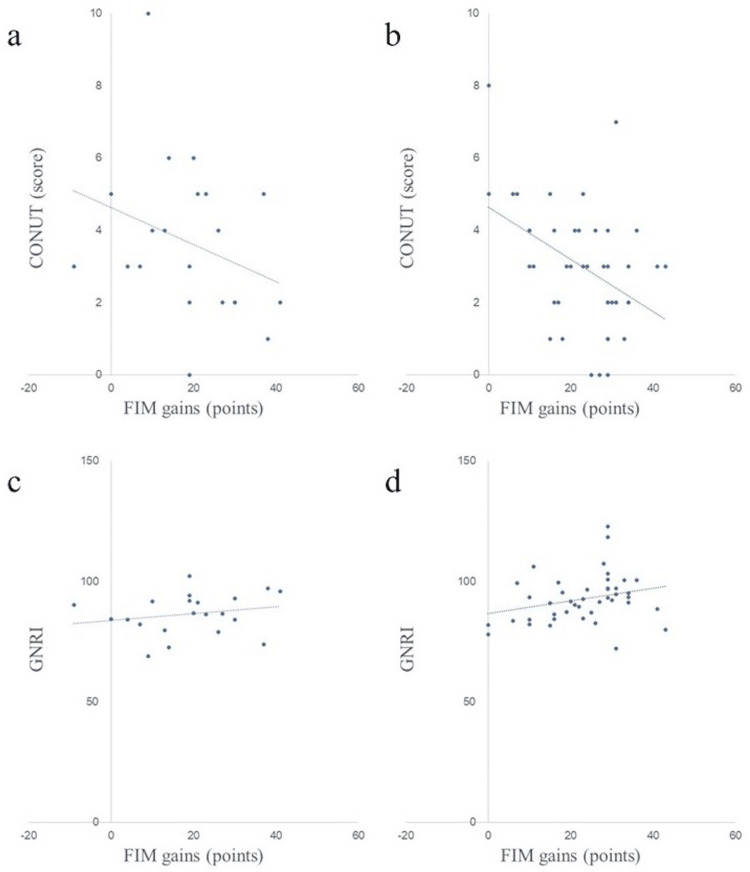
Scatter plot of nutritional evaluation items (CONUT and GNRI) at admission and FIM gains. (a) CONUT at admission and FIM gains in the super-elderly group. (b) CONUT at admission and FIM gains in the control group. (c) GNRI at admission and FIM gains in the super-elderly group. (d) GNRI at admission and FIM gains in the control group. CONUT = Controlling Nutritional Status; GNRI = Geriatric Nutritional Risk Index; FIM = Functional Independence Measure

**Figure 5:**
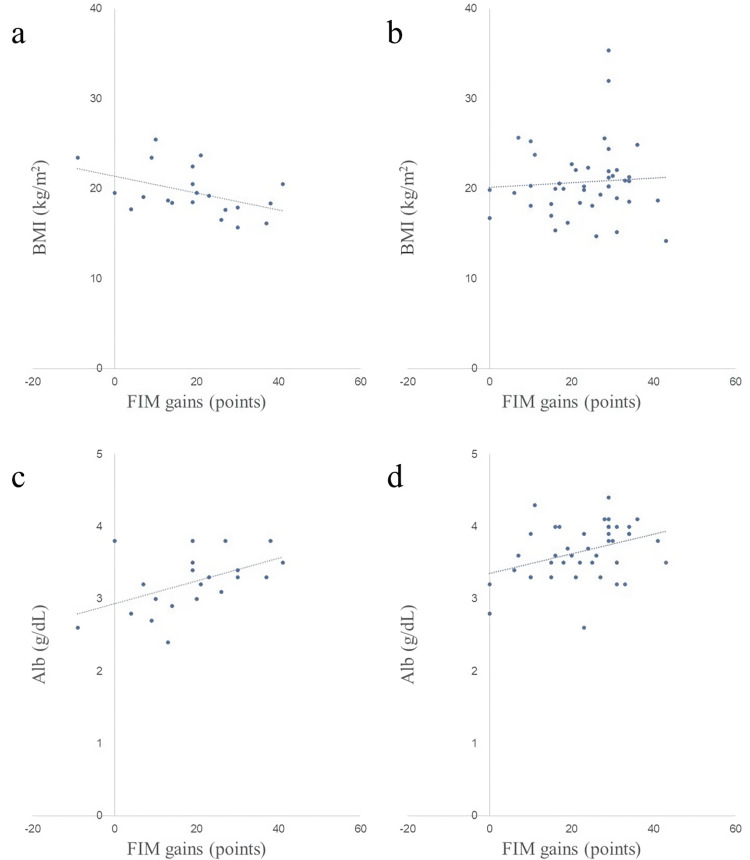
Scatter plot of nutritional assessment items (BMI and Alb) at one month post-hospitalization and FIM gains. (a) BMI at one month and FIM gains in the super-elderly group. (b) BMI at one month and FIM gains in the control group. (c) Alb at one month and FIM gains in the super-elderly group. (d) Alb at one month and FIM gains in the control group. BMI = body mass index; Alb = serum albumin; FIM = Functional Independence Measure

**Figure 6:**
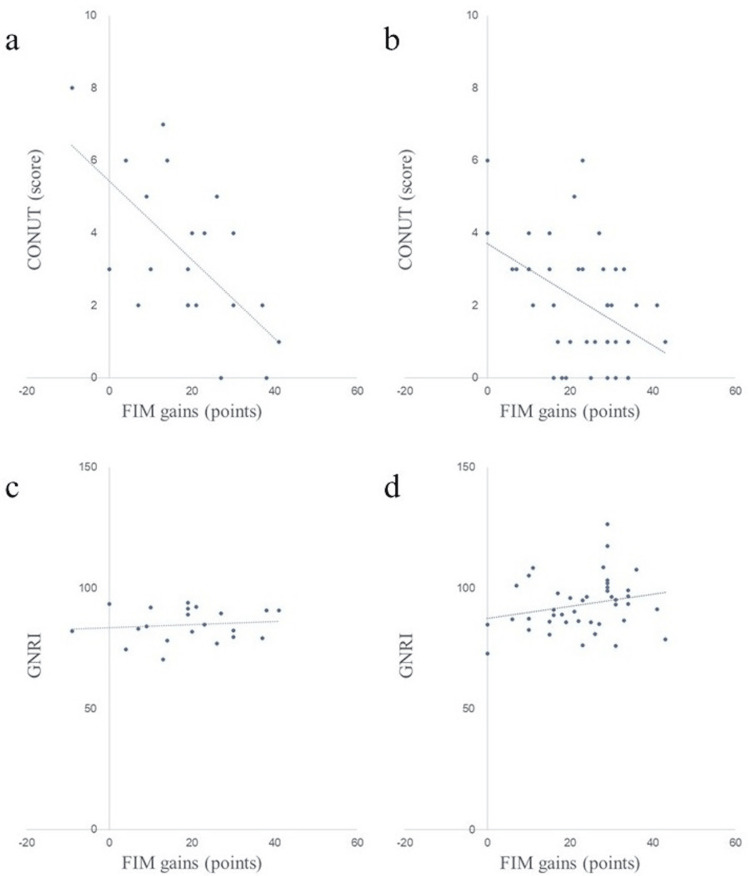
Scatter plot of nutritional assessment items (CONUT and GNRI) at one month post-hospitalization and FIM gains. (a) CONUT at one month and FIM gains in the super-elderly group. (b) CONUT at one month and FIM gains in the control group. (c) GNRI at one month and FIM gains in the super-elderly group. (d) GNRI at one month and FIM gains in the control group. CONUT = Controlling Nutritional Status; GNRI = Geriatric Nutritional Risk Index; FIM = Functional Independence Measure

**Figure 7:**
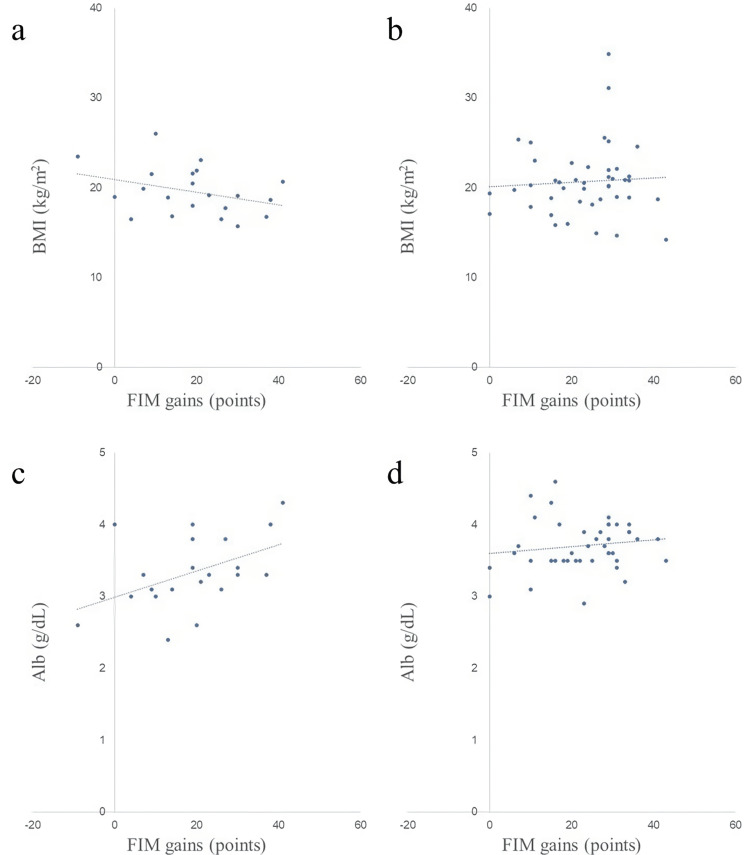
Scatter plot of nutritional assessment items (BMI and Alb) at the final evaluation and FIM gains. (a) BMI at the final evaluation and FIM gains in the super-elderly group. (b) BMI at the final evaluation and FIM gains in the control group. (c) Alb at the final evaluation and FIM gains in the super-elderly group. (d) Alb at the final evaluation and FIM gains in the control group. BMI = body mass index; Alb = serum albumin; FIM = Functional Independence Measure

**Figure 8:**
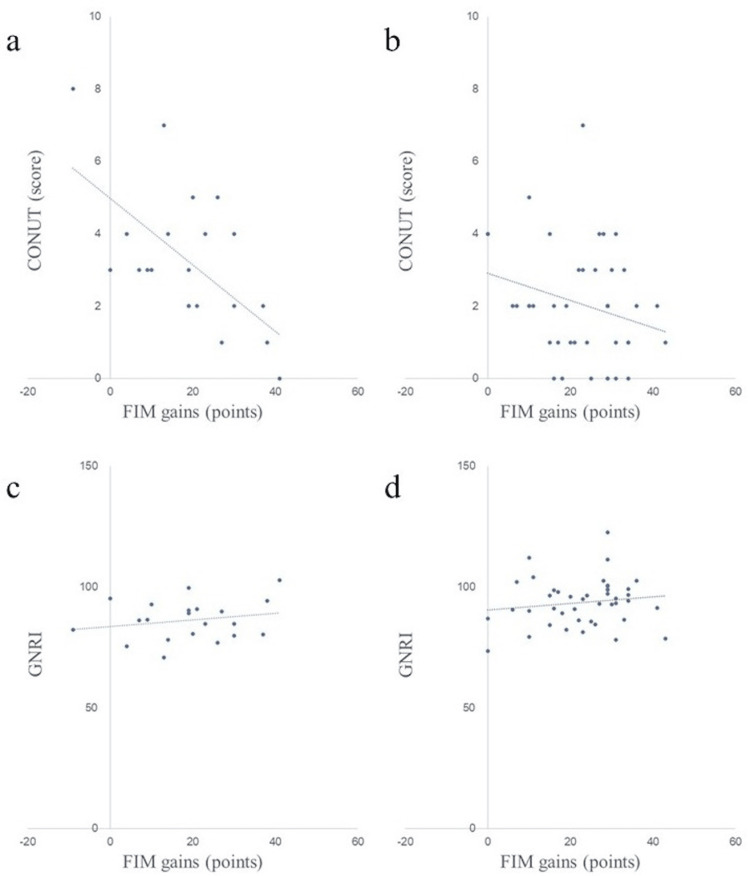
Scatter plot of nutritional assessment items (CONUT and GNRI) at final evaluation and FIM gains (a) CONUT at the final evaluation and FIM gains in the super-elderly group. (b) CONUT at the final evaluation and FIM gains in the control group. (c) GNRI at the final evaluation and FIM gains in the super-elderly group. (d) GNRI at the final evaluation and FIM gains in the control group. CONUT = Controlling Nutritional Status; GNRI = Geriatric Nutritional Risk Index; FIM = Functional Independence Measure

Scatter plots of nutritional assessment items and FIM gains in both groups are shown in Figures 2-8. Figures 2-4 are scatter plots showing nutritional assessment items at admission and FIM improvement levels. Figures 5, 6 are scatter plots showing nutritional assessment items one month after admission and the increase in FIM scores. Figures 7, 8 are scatter plots showing nutritional assessment items at final evaluation and the gain in FIM scores. Results of the correlation analysis are shown in Table 3, and correlation coefficients for items with significant correlations are presented in Table 4.

**Table 3:** Correlation between nutritional evaluation items and FIM gains. *: p < 0.05 statistically significant; p < 0.01 statistically significant. MNA-SF = Mini Nutritional Assessment-Short Form; BMI = body mass index; Alb = serum albumin; CONUT = Controlling Nutritional Status; GNRI = Geriatric Nutritional Risk Index; FIM = Functional Independence Measure

	Evaluation items	Super-elderly group (n = 21)	Control group (n = 43)
At admission	MNA-SF	0.53	0.117
	Calf circumference	0.30	0.422
	BMI	0.44	0.419
	Alb	0.05*	0.041*
	CONUT	0.129	0.009**
	GNRI	0.192	0.037*
At one month	BMI	0.041*	0.531
	Alb	0.02*	0.023*
	CONUT	0.005**	0.003**
	GNRI	0.877	0.107
At the final evaluation	BMI	0.206	0.503
	Alb	0.033*	0.294
	CONUT	0.015*	0.113
	GNRI	0.546	0.318

**Table 4:** Correlation coefficient between nutritional evaluation items and FIM gains. The values are presented as correlation coefficients (95% CIs). Guilford (1956) definition: 0 < r < |0.2| indicates no significant correlation, |0.2| < r < |0.4| indicates weak correlation*, |0.4| < r < |0.7| indicates moderate correlation**, |0.7| < r < |1| indicates strong correlation***. MNA-SF = Mini Nutritional Assessment-Short Form; BMI = body mass index; Alb = serum albumin; CONUT = Controlling Nutritional Status; GNRI = Geriatric Nutritional Risk Index; FIM = Functional Independence Measure; CI = confidence interval

	Evaluation items	Super-elderly group (n = 21)	Control group (n = 43)
At admission	MNA-SF	-	-
	Calf circumference	-	-
	BMI	-	-
	Alb	-	0.313 (CI: 0.014, 0.561)*
	CONUT	-	-0.392 (CI: -0.620, -0.104)*
	GNRI	-	0.319 (CI: 0.020, 0.565)*
At one month	BMI	-0.449 (CI: -0.738, -0.022)**	-
	Alb	0.505 (CI: 0.093, 0.769)**	0.347 (CI: 0.052, 0.586)*
	CONUT	-0.587 (CI: -0.813, -0.209)**	-0.437 (CI: -0.652, -0.158)**
	GNRI	-	-
At the final evaluation	BMI	-	-
	Alb	0.466 (CI: 0.043, 0.747)**	-
	CONUT	-0.525 (CI: -0.780, -0.121)**	-
	GNRI	-	-

In the control group, CONUT (correlation coefficient (r) = -0.392), Alb (r = 0.313), and GNRI (r = 0.319) scores upon admission and CONUT (r = -0.437) and Alb (r = 0.347) scores after one month were significantly correlated with FIM gain. In the super-elderly group, no significant correlation was found between FIM gain and any nutritional assessment item upon admission, but significant correlations were found between FIM gain and CONUT score (r = -0.587) and Alb level (r = 0.505) at one month after admission and between FIM gain and CONUT score (r = -0.525) and Alb level (r = 0.466) at the final assessment. Additionally, in the super-elderly group, the CONUT one month after hospitalization was strongly correlated with FIM gain. BMI one month after hospitalization in the super-elderly group was negatively correlated with FIM gain.

## Discussion

In super-elderly patients, nutritional indices at admission were not associated with improvements in ADLs. However, CONUT scores and Alb levels measured one month after admission and at the final evaluation showed significant correlations with improvements in FIM motor function. A significant correlation was observed between FIM gain and CONUT score, Alb level, and GNRI upon admission, and CONUT score and Alb level after one month in the control group (aged 75-89 years). Conversely, in the super-elderly group (aged ≥90 years), no significant correlation was found between FIM gain and each nutritional assessment item at admission, but a correlation with FIM gain was observed in CONUT and Alb level after one month, and CONUT and Alb at the final assessment.

Regarding nutritional assessment items, the MNA-SF is used for nutritional screening of elderly people aged ≥65 years, with scores of 0-7 indicating malnutrition, 8-11 indicating risk of malnutrition, and 12-14 indicating good nutritional status [13,19]. Here, the median MNA-SF score upon admission was 6.0 (5.0-7.0) and 6.0 (5.5-7.5) points in the super-elderly and control groups, respectively, with both groups falling into the category of at risk for malnutrition. Calf circumference was used as an indicator of lower leg muscle mass, with cutoff values <33 cm for men and <32 cm for women, as recommended by the Global Leadership Initiative on Malnutrition (GLIM) [20]. The median calf circumference was 27.0 (25.0-29.0) and 29.8 (27.9-32.5) cm in the super-elderly and control groups, respectively, both of which were below the cutoff value. Furthermore, significant differences in calf circumference were observed upon admission, with lower values in the super-elderly group, suggesting reduced lower limb muscle mass. BMI was used as a criterion for moderate malnutrition in the GLIM criteria, with a BMI <20 kg/m² for individuals aged ≥70 years [20,21]. The median BMI was 19.4 (17.9-21.0) and 21.1 (18.7-22.7) kg/m² in the super-elderly and control groups, respectively, with the super-elderly group falling below the criteria. Additionally, according to the Ministry of Health, Labour and Welfare’s Japanese Dietary Intake Standards [22], the target BMI range for the elderly is 21.5-24.9 kg/m², and both groups fell below this target range. The same trend was observed for BMI at one month after admission and at the final evaluation. The CONUT is a nutritional assessment method with high concordance with the Subjective Global Assessment, which scores values obtained from blood biochemical tests (Alb, total lymphocyte count, and total cholesterol) to classify patients into four categories, i.e., 0-1 point (normal), 2-4 points (mild malnutrition), 5-8 points (moderate malnutrition), and ≥9 points (severe malnutrition) [23]. CONUT does not use physical examination findings; therefore, it is recommended in conjunction with clinical information [24]. The median CONUT score upon admission was 3.0 (2.0-5.0) and 3.0 (2.0-4.0) in the super-elderly and control groups, respectively, both of which were classified as mild malnutrition. The CONUT score was 3.0 (2.0-5.0) and 2.0 (1.0-3.0) one month after admission and 3.0 (2.0-4.0) and 2.0 (1.0-3.0) at the final evaluation in the super-elderly and control groups, respectively, both of which corresponded to mild malnutrition.

The GNRI is a predictive indicator for the incidence of complications and mortality in elderly individuals aged ≥65 years and has been reported to be useful for assessing the nutritional status of hospitalized elderly patients [16]. The GNRI classifies patients with a score <82 as having a high risk, those with a score of 82-91 as having a moderate risk, those with a score of 92-97 as having a low risk, and those with a score ≥98 as having no risk. The median GNRI upon admission was 86.6 (82.2-91.9) and 92.2 (85.4-97.0) in the super-elderly and control groups, respectively, with the super-elderly group classified as moderate risk and the control group as low risk. The GNRI was 84.2 (79.8-90.7) and 93.2 (86.0-99.0) one month after admission and 86.1 (80.3-90.9) and 94.2 (86.7-98.9) at the final evaluation in the super-elderly and control groups, respectively.

Alb, which accounts for approximately 50% of the total serum protein, has been used as a nutritional assessment parameter. However, it increases in response to steroids, insulin, thyroid hormones, and dehydration and is influenced by inflammation, metabolic hyperactivity, severe liver or kidney dysfunction, malabsorption, excessive circulating blood volume, and half-life; therefore, caution is necessary [24-26]. Recently, Alb has been recognized as an inflammation indicator rather than a nutritional indicator [27,28]. In this study, inflammatory markers such as C-reactive protein levels were not measured concurrently with nutritional assessment. Measuring inflammatory indicators when interpreting Alb levels is considered a topic for future research. The reference range for Alb is 4.0-5.0 g/dL, and in CONUT, Alb levels <3.5 g/dL are considered a nutritional risk. The median Alb level upon admission was 3.3 (3.0-3.8) and 3.6 (3.3-3.8) g/dL in the super-elderly and control groups, respectively, with the super-elderly group falling below 3.5 g/dL. The Alb level was 3.3 (3.0-3.5) and 3.7 (3.5-4.0) g/dL one month after admission and 3.3 (3.1-3.8) g/dL at the final evaluation in the super-elderly and control groups, respectively. All nutritional assessment parameters in the super-elderly group met the malnutrition criteria at all time points.

In the super-elderly group, CONUT and Alb scores correlated with FIM gain after one month and at the final evaluation. The criteria for the strength of the correlation coefficients were based on Guilford (1956), who defined the following ranges: 0 < r < |0.2| indicates no significant correlation, |0.2| < r < |0.4| indicates weak correlation, |0.4| < r < |0.7| indicates moderate correlation, and |0.7| < r < |1| indicates strong correlation [29]. The CONUT score includes Alb, total lymphocyte count, and total cholesterol levels, as described previously. Regarding total lymphocyte count, a decrease to 900-1,500 per mm³ indicates moderate malnutrition, and a count ≤900 indicates severe malnutrition [23]. Total cholesterol level is reportedly low in patients with nutritional disorders or severe liver parenchymal disease [24]. As with Alb, the total lymphocyte count and total cholesterol levels may vary due to factors besides nutritional status; therefore, these factors must be considered. The CONUT score one month after hospitalization correlated with FIM gain, particularly in the super-elderly group, suggesting an association between the CONUT score one month after hospitalization and ADL improvement through rehabilitation treatment. The rationale is that postoperative catabolism and early inflammation may mask the true nutritional status at admission. Furthermore, the half-life of Alb is approximately three weeks. In the super-elderly group, stabilization of inflammation and improvement in nutritional status were reflected in nutritional assessment items after one month of hospitalization, and a correlation with improved ADLs was presumed.

This study found a negative correlation between BMI and FIM gain after one month in the super-elderly group. No significant relationships were found for the other BMI items. As many factors other than nutrition affect weight gain or loss, it is necessary to examine the factors that cause weight changes. Only one patient in the super-elderly group showed a decrease in FIM gain and weight loss. This case involved a patient who received oral intake and an intravenous drip for four weeks during hospitalization. As super-elderly individuals are likely to develop dysphagia and loss of appetite, caution is required in discussing their nutritional status based on BMI.

Previous studies have reported that nutritional status upon admission to a convalescent rehabilitation ward is associated with FIM scores upon admission and discharge and improvements in those scores [10-12]. Here, the control group showed a significant correlation between the nutritional assessment items upon admission and FIM gains. However, in the super-elderly group, a correlation between FIM gain and CONUT and Alb scores one month after admission was observed, but not with nutritional assessment items upon admission. The study results differ from those of previous studies, suggesting that the relationship between nutritional status and ADL improvement in super-elderly patients with proximal femoral fractures may differ from that in elderly patients aged 75-89 years (control group). Furthermore, in super-elderly patients with proximal femoral fractures, CONUT score and Alb level one month after hospitalization were associated with improvements in ADL following rehabilitation treatment. Therefore, super-elderly patients may require active nutritional improvement from the initial stages of rehabilitation hospitalization. As a pragmatic protocol, it is recommended to perform screening tests upon admission, followed by a routine reassessment one month later. Persistently elevated CONUT (≥3) or Alb (<3.5 g/dL) may trigger enhanced nutritional management alongside rehabilitation. In the control group, many items met the criteria for malnutrition, similar to previous studies. Therefore, we believe that proactive efforts to improve nutrition are necessary for elderly individuals aged 75-89 years.

This study had several limitations. First, it was conducted at a single institution with a small sample size; therefore, future studies involving multiple institutions should be conducted. Second, as this study did not investigate the causes of malnutrition and inflammation, or the effects of medical history and comorbidities, these factors should be examined in future studies. Recently, Alb has been reported to be an inflammation indicator rather than a nutritional indicator [27]. In this study, improvements in inflammation and nutritional status may have contributed to improvements in ADLs. It is necessary to monitor nutritional status and inflammatory markers over time. Third, muscle mass was not evaluated in this study. Nutritional assessment based on regular muscle mass evaluation [30,31] is not always possible in medical facilities, which is an issue that should be addressed. Finally, this study was conducted without risk adjustment for multiple comparisons to clarify the characteristics and trends in nutritional status and ADL improvement among the super-elderly patients. Moving forward, to develop this research and perform more detailed analyses, it will be necessary to consider statistical methods that account for the risk of multiple comparisons. We would want to further develop this research by reviewing malnutrition screening methods, nutritional assessment items, and regular assessment methods to improve the quality of nutritional interventions.

## Conclusions

We investigated the association between nutritional status and improvement in ADLs in super-elderly patients who underwent rehabilitation therapy in a rehabilitation ward following surgery for proximal femoral fractures. A total of 21 patients in the super-elderly group, aged 90 years or older, and 43 patients in the control group, aged 75 to 89 years, were included in this study. The following nutritional assessment items were extracted from medical records: MNA-SF, calf circumference, BMI, Alb, CONUT, and GNRI. The correlation between FIM gain (difference in total FIM motor item scores between admission and discharge) and the nutritional assessment items was analyzed. In the super-elderly group, no significant correlation was found between nutritional assessment items at admission and FIM gains. However, CONUT and Alb values at one month post-admission were associated with ADL improvement. Our findings suggest that nutritional management after hospitalization is associated with improved ADL in super-elderly patients, but prospective validation is required.
